# Connecting Mindfulness Practice to Generative Semiotic Activity—Self-Other Referent in Umwelt and Semiosphere

**DOI:** 10.1007/s12124-024-09851-x

**Published:** 2024-07-17

**Authors:** Masayoshi Morioka

**Affiliations:** Ritsumeikan University, Kyoto, Japan

**Keywords:** Mindfulness, Mead, Semiotic activity, Buber, Rogers, Umwelt, Semiosphere, Tao, *I Ching*, Mind and body, Non-human, Referent, Interoception, Tonus

## Abstract

**Abstract:**

In this comment paper on von Fircks (2023a), I would like to focus on four issues and offer some reflections on them: first, what is happening in the process of a new I arising through mindfulness meditation practice? I would like to supplement the dialogue between Buber and Rogers in 1957 on the dynamism of I and Me, which is the basis of Mead's theory of self formation, in which I and Me separate, discover and meet a new self. The second, is that meditation, which at first glance appears to be an internal meditation practice and a personal activity, leads to a semiotic mediated social process. The Tao and early Buddhist ideas that form the background to the experiential process of mindfulness meditation will be reviewed, and the significance of people experiencing the interdependence of non-human nature and the environment through the practice will be discussed. Third, connecting this to the idea of Umwelt (Uexküll) and the semiosphere (Lotman), an attempt is made to extend the otherness as a collating body of self formation to Umwelt. Fourth, mindfulness meditation focuses attention on the breath. In relation to Mead's focus on the environment under the skin, i.e. corporeality, I will supplement the psychological meaning of cultivating the body's sense of interoception through the sensing of repetitive movements of tension and relaxation. Through the above, what kind of semiotic mediating function does mindfulness meditation have in relation to the construction of the new I, and how does it lead to the creation of social meaning? We would like to discuss these points.

**Clinical Trial Registration:**

The article does not contain any studies with clinical trial. This, clinical Trial registration is not applicable.

I and Me

「寒いね」と話しかければ「寒いね」と答える人のいる あたたかさ　俵万智

(Warmth, with people who say, “It’s cold.” when you talk to them, “It’s cold, isn't it?”. Machi Tawara Japanese *Tanka*)

The subject of von Fircks (2023a), in which an apparently personal, internal meditation discipline leads to a social system as a semiotic mediated process, is original and practically significant. In particular, it offers a new perspective on overcoming the psychosocial crisis in the self-formation of modern man. The last sentence of von Fircks' conclusion is fascinating. “In short, meditation alters powerfully semiotic mediation and thus the ways how we interact with our social system.(my emphasis)”.

Through mindfulness meditation practice a new I arise. von Fircks (2023a) presented a process of self-rediscovery based on autoethnography. In elucidating the process, von Fircks uses Mead's self theory (Mead, 1934/2015). The self has two aspects. The I is not clearly manifested in self-consciousness; it is the Me that clearly appears in consciousness. However, Me cannot exist without I. For example, when we write, we write while thinking, i.e. while talking to ourselves. What we write is Me, but we are often unaware of the I that carries out the writing. Often, when we see what we have written, we are surprised at the finished product. I am even surprised to wonder who wrote it and how it was written, even though it is my own work. The I am doing work that goes beyond what I intended beforehand, but I don't realize it. Rather, I know after the fact. In the execution process of writing, I is in the background and does not stay in the consciousness. On the other hand, Me comes to the front of the situation, including the act; The I responds to it, but moves in an indeterminate way. Mead considers the self to be a social process that proceeds with these two aspects.

The position of others in the process is important. Mead emphasizes the importance of speech gestures in the development of the mind. Speech gestures evoke the same style of response in the individual's own mind as they evoke in others. One can place oneself in the position of the Other by hearing one's own voice with one's own ears at the same time as one's own voice is heard by the Other and evokes a response. A perspective arises in which one takes on the role of the Other within oneself.

The self becomes its own object. How is it possible to make oneself an object, as many painters leave self-portraits? Through looking out from oneself and looking at oneself, one forms one's self. It is the experience that arises when he/she becomes an object to himself/herself, just as others see him/her, that makes the self. How does this happen? The first is that the self takes on itself the stance that others take towards it. This is done by diving into the perspective of the other. And it is through the semiotic mediation of speech gestures that it is possible to place oneself in the position of the other. Meaning arises when the expected outcome between people is accompanied by gestures. And introspective meaning arises there. Mead sees the sense of meaning that arises from this responsive relationship as the starting point of the mind.

Mead does not take the idea that the self comes first and then it relates to others. The self is constituted through the process of communication with others and the act of communication. This is Mead's basic idea. The individual works not only with others, society and the natural environment, but also with himself. This is possible through semiotic mediation processes. In particular, the emphasis on gestural conversation is an original part of Mead's work.

## Self in Rupture and Encounters

What kind of communication characteristics does the practice of mindfulness meditation have? Through the communication process, a new I is created. This process is depicted by von Fircks (2023a) as the experience of mindfulness meditation, but what social meaning-making processes are involved? Further exploration is needed.

In the self formation as a dynamic process of subject I and object Me, how are others involved in it? According to Mead, Me is not only the object of I, but also emerges as the object of the Other. The I is the part of the Other that sees Me as Me. In this sense, it is open to exchange with the Other. And through the double mediating process of signs, the inner dialogue is activated. Here, the issue of the dialogicality of human consciousness lies in the background. The present state of the subjectified subject is woven into the future state of the unsubjectified subject. This is made possible through the work of signs. People change the social signs that enable them to communicate with others in individual ways according to the social situation and the needs of others. This process connects the present and the future.

On the other hand, I and Me are not always harmonious. Sometimes ruptures occur; it is quite possible that I and Me do not come to terms and clash. Sometimes Me rise up in front of I as a wall. von Fircks focuses on the ruptures that arise between I and Me. It can be a source of conflict and suffering for people. Then Me meets a new I. Therein lies the healing. The two I's who have developed a rupture meet again. In accordance with Mead's psychology, I emerges through a person's response to a social situation, and I moves towards Me, which is formed by the response to others, and realizes Personality. Personality is the higher order of society.

The process of detachment and encounter between I and Me is also a theme that is linked to psychological maladjustment and the process of recovery. Here, I would like to introduce as an auxiliary line of argumentation to von Fircks (2023a), an argument that was sharpened by the dialogue between Rogers and Buber, which laid the foundations for counselling theory. Like Mead, they were thoroughly practical about how the self is formed in relation to others.

## Buber and Rogers in Dialogue

In psychology and psychiatry, Martin Buber (1878–1965) is well known and his philosophy of dialogue has had a major impact following the publication of “I and Thou” in 1923. In his later years, Buber held a dialogue with Carl Rogers (1902–1987), a counselling psychologist, at the University of Michigan, Ann Arbor, on 18 April 1957. As a historical dialogue in psychology, the precise recording has been mined and published 40 years later (Anderson & Cissna, 1997). The dialogue revolved around why dialogue heals people and the healing work of the I-thou relationship. Moments of 'true encounter' occur between the client and the therapist during a counselling interview. Rogers draws on this experience and states that it approaches an 'I-thou' relationship. Buber, on the other hand, maintains that the therapeutic relationship is not equal and that the various constraints that exist should be taken up one by one and the situation and reality should be faced.

The moment when a person change is the moment when the relationship is experienced in the same way on the part of both therapist and client (Rogers, 1951, p, 53). In such moments, we can clearly sense how the person's experience is received by the person himself/herself. We can see the person's experience from within the person. These decisive moments that occur in psychotherapy, which Rogers calls 'true encounters'. One of the important ways of being in relation to encounters and relationships is in a person's relationship to himself or herself. 'It is at that moment of encounter with oneself, perhaps precisely at that moment, that a person can truly meet another person in an I-thou relationship'. This question of Rogers is fundamental to self-relationality and reflection in therapy.

Now, we become aware of a new part of the self and encounter the self as such. In the framework of Mead's self theory, this would mean discovering a new I out of the I and the Me that I have captured, but Rogers would rather mean that the I encounters a new I. It refers to an experience similar to what von Fircks (2023a) refers to as the encounter with the new I that occurs through mindfulness meditation. Buber is negative on whether he would call it a dialogue. Surprise in the encounter with the self certainly occurs. But surprise in relation to the Other, Buber states, is quite different from surprise in the inner encounter.

Through mindfulness meditation, von Fircks (2023a) encounters the other I as if it were an encounter with another person. He is guided by surprise in the encounter. Rogers also identified noticing and deepening the encounter with oneself as one important type of dialogue. Like meeting an almost stranger, one encounters one's own experience, which one has never encountered before. This is where the surprise lies, he states. Clients are 'suddenly struck by the meaning of something that has arisen from somewhere within him, from somewhere he cannot even recognize. He is surprised by his very self" (Anderson & Cissna, 1997, p.72). Thus, Rogers states with conviction.

## Diving Through the Narrow Passage of the Other

Encountering the self and dialogue with the self are familiar themes often used in the practice of psychotherapy, but true dialogue as described by Buber differs from dialogue with the self in that the otherness of the Other is an issue. In his article ' Das Wort, das gesprochen wird ' (1960), after the dialogue with Rogers, Buber examined this issue again from the perspective of who hears the word (Buber, 1960). The so-called dialogue with the self is made possible by the fundamental fact that people talk to each other. Their internalization is a dialogue with the self. Referring to this, he states that dialogue within the self is possible only if the dialogue exchange with the other, which is accompanied by a moment of surprise, comes first. Surprise at the otherness is an inevitability that accompanies the process of dialogue. By keeping this in mind, the space for dialogue is secured (Anderson, 1997; Morioka, 2011).

Indeed, the individual self fluctuates. The self is seen as a distributed, fluid, relational and allocated according to place (Monk & Zamani, 2019). The attempt by von Fircks (2023a) to depict and analyze the points at which these characteristics of the self emerge through the practice of mindfulness meditation is an ingenious attempt. Although from a completely different standpoint, Myerhoff's practice is pioneering (Myerhoff, 1992). The practice of small group storytelling and listening to an individual's life also had its roots in Definitional Ceremony of narrative practice (White, 1995). Through the act and performance of group storytelling and listening, a scene is set up like a dramatic stage. Each participant's experience is initially raw chaos in itself. When one's own narrative is received by others in the group, it becomes possible to carve a meaningful coherence out of the chaos with their support. Through this act of storytelling, the me who tells becomes clearer than I was before. By telling each other personal histories and making them visible, the narrative is transformed into an experience. The self becomes certain. Meyerhoff called this collective activity of self-definition through having others there to hear and receive you Definitional Ceremony.

## Tao’s View of the World

It is considered unique to mankind that communication between oneself and others and communication between oneself and others is a coupling relationship and can be simultaneous. Only people can use the communication code of coupling (Sebeok, 1972). However, dialogical communication is not only concentrated between people and people, but also people and organisms, people and objects. The way we communicate and interact with the environment around us is also deeply related to the formation of the human self. The interaction between human and non-human beings should not be forgotten. It is the Tao and early Buddhist ideas that form the background to the experiential process of mindfulness meditation, which aim to experience the interdependence of nature, environment and people through the practice. It is worth noting here.

Wilhelm's translation of Jung's detailed annotations to the book, *The Secret of the Golden Flower*, was published in 1929 and had a wide influence on Western thought in the 1930s. Hermann Hesse was one of them. And there is a direct link to Jung's psychological thought. The author translates inaction; 無為 in Tao as the art of not-forcing, (not non-doing). This perspective is important. We assume that the author has explored these areas through his study of Hesse’s late works (von Fircks, 2022). The Tao, or Lao-Zhuang view of the world, holds that man's true nature is to be united to the great life of the universe, and that the ideal is a life of inaction and nature. The ideal is to cut off worldly cares and to immerse oneself in nature in the naked form. There, man becomes one with nature. The Tao is dedicated to this cause, but the source of the Tao is *I Ching*. As Wilhelm's German translation translates it as the *Book of Changes*, it sees nature, the world and human life as constantly in flux.

*I Ching* is the practice of semiotic interpretation, the reading of changes in heaven and earth by means of sixty four hexagrams. It is a philosophy that integrates two ideas: yin-yang and the five elements. In Yin-Yang, a pair of opposites, Yin and Yang, are repeatedly generated and annihilated, from which all things arise. In the Five Elements,—wood, fire, earth, metal and water—mutually influence each other and repeat generation and extinction in a 'symbiosis/concurrence', similar to yin and yang. Thus, the foundation of both philosophies is a worldview in which individuals (parts) are organically connected to form a unified body (system), within which each individual undergoes endless and repeated change.

The key factor here is the existence of time. The fact that generation and extinction are repeated means that time exists there and that changes occur with the flow of time. The flow of time circulates in a circle, and the changes that occur there never cease. However, a circle does not mean that it returns to its original point. This is because change exists in the passage of time. This concept of change and time is important in understanding the Yin Yang and the Five Elements. The *I Ching* reads change, as it is called the 'Book of Changes' (1967). It also teaches us to read the signs of change.

Now, the author is original in reading I Ching as an inner-self dialogue. Mindfulness meditation is the source of the essence of early Buddhist practice: how does Tao connect with early Buddhist theories and ideas? Jung's lectures on the psychology of Eastern spirituality on late1930’s took a comprehensive view of the religion of Tibet, India, Zen, *Jodo*, 浄土, *I Ching* and attempts to address the problems of the modern human soul from both Eastern and Western perspectives.

Buddhism, which is the source of mindfulness meditation, is originally about knowing oneself, but the path to knowing oneself is often difficult. The manners and methods to achieve this have been passed down orally since ancient times. The simplest style is probably mindfulness. The difficulty of knowing oneself and the mind's attachment to people and things is endless. The suffering that arises from this, especially birth, illness, old age and death, is not at the mercy of the individual. Suffering is the starting point of Buddhism. We encounter unreasonable disasters. Why do I have to go through this? There is no answer to this why. When you are faced with a problem for which there is no answer, you need an inner Other who is willing to listen to you. This communication is the first step, which is at the same time a dialogue with the self, a place of introspection.

## Interdependence of Mind and Body

Both Buddhism and Tao advocate mind-body oneness and consider mind and body to be inseparably related. In psychosomatic medicine, too, it is a clinical fact that the mind-body relationship cannot be explained by causal theory. What happens in the mind also causes some changes in the body. The reverse is also true. A different logic from causal theory would be required. Namely, 'when one event phenomenon A arises in the mind, another event A' arises in the body. Wherein there is a functional correspondence between them" (Nagatomo, 2014) The arising of A is grasped from the side of the mind and from the side of the body. It is not one or the other. It is neither. It is both. Describing such phenomena requires a logic other than causal or binary logic. How do we describe a phenomenon that is both neither and both?

One clue is an attempt to view mind and body as a dependent relationship, based on the Buddhist concept of auspiciousness; 縁起. When this (the mind) occurs, that (the body) occurs. Such interdependence occurs between this and that; this and that do not belong to either mind or body. Here, the interdependence de-emphasizes causality (Nagatomo, 2014; Morioka, 2021).

This leads to the idea of placing the third term (the third) between this and that. Nagatomo sees the third term as the source of life energy (*Ki*: 気), and Tao is a technique of touching the *Ki* that fills the body-mind-natural environment and self-personal relationships, taking it into one's own guidelines, cultivating it and working with it in all directions. It is also possible in practice to incorporate the relational theory of the body in dialogue. The mind-body correlation can be explained on the basis of the logic of karma, but Tao attempts to express it dynamically by incorporating the third term into it. The function of this third term, *Ki*, is in close proximity to the meaning-generating activity of signs. Morioka (2011) also analyzed the characteristics of the field of semiotic activity using the space-time concept *Ma*; 間, which is unique to Japanese culture, as the third term.

## Umwelt and the Semiosphere

Let us return to Mead's theory of the self. The starting point of Mead's thought is the reality of the perspectives of the Other. The individual enters the perspectives of the Other insofar as he or she is able to assume the stance of the Other, the perspective of the Other. When I stand in the other's perspective, the other's self enters my experience. And when the experience of the other becomes his own object, it enters into his own experience. At this point, no boundary can be drawn between my own self and the self of the other (Mead, 1934/2015, p.175).

Here, I would like to extend the perspective of the self's referents, the otherness, to the environment. Rather than restricting the other to a specific person (generally a significant other for the individual), how do the perspectives of the other appear in the ecological environment of the person, which the self refers in shaping itself, and how do they mediate the encounter with the new I?

Mead theorizes about the interrelationships between life forms and the environment. It is a kind of forerunner of modern ecological environmental theory. Various forms of life shape the environment. And organisms are, in a sense, responsible for their environment. And since the organism and the environment are mutually regulating and interdependent for their existence, a proper understanding of the life process must be considered in terms of their interrelationships (Mead, 1934/2015, p. 130).

More specifically, Mead describes the animal's relationship to its food-'object' as follows. When an animal comes into the world that can digest grass, like a cow, then grass becomes food. The object did not exist before, i.e. grass as food did not exist. With the appearance of the cow, a new object is brought into existence. In this sense, the organism is responsible for the emergence of a series of objects that did not exist before (Mead, 1934/2015, p. 129). In other words, 'only when the cow is present does the grass become food'. Mead discusses the interrelationship between organisms (life forms) and their environment in this way.

Examining the area where the self and the environmental world intersect, we are constantly influenced by and working with our environment. In other words. The Umwelt introduced by Uexküll (1909/1970) is not an external environment seen from a person-centered perspective. Uexküll envisages a biology that is concerned only with the living forms of life and sees the organism as a total entity embedded in Umwelt. The organism is an active being that performs as a role-player in each life situation. Living organisms are subjects that form the center of the semiotic world (Mittelpunkt der Bedeutungswelt), each of which forms its own Umwelt. If the world is depicted from this perspective from the point of view of living organisms, the view of life is that living organisms form an infinitely diverse parallel world, and that there is no superiority or inferiority between the worlds of sea urchins and humans. The world as a whole is like the sum of an orchestra.

From the point of view of Uexküll, life forms, not just people, make sense of Umwelt. Every living thing on the planet survives as part of a huge set of ecosystems, the biosphere, and everything that the organism perceives has meaning for it. That meaning can be food, escape, reproduction or disappointment in it. For a living organism, adaptation to it is a condition for survival. In this sense, each living organism can be described as living in a semiotic world.

Lotman (1990) named it the semiosphere. The semiosphere the totality of individual semiotic phenomena (text, language, communication) and at the same time a semiotic space that is the condition for their existence and functioning. Considered as an environment, the semiosphere refers to a region of the earth similar to the atmosphere, hydrosphere and biosphere. The semiosphere is a large sphere, which penetrates into every other sphere and extends to every corner of it, integrating sounds, smells, gestures, colors, shapes, electric fields, thermal radiation, all waves, chemical signals, contact and all other kinds of communication. In a word, it is all the signs of life. All life on earth is born into this sphere of signs. In the case of humans, in addition, there is a semiotic space specific to a given culture, the whole of which functions as one. This is called the semiosphere.

## Semiotic Activities and Affect

For people, Umwelt is not identical to the external environment surrounding them. Umwelt is life, life itself. And the sensory stimuli that a person perceives in the environment are constrained by the workings of the inner world. It selects and arranges the stimuli of the external world. The basis of this inner activity is emotion and desire or affect. Semiotic mediation is at work when the self forms a new self via the perspective of the other, and this process involves affect. Although we tend to assume that semiotic activities are rich and creative, the opposite is rather the case in various psychosocial support situations. Stimuli with strong emotions, such as violent words or actions from others or unforgivable failures, are planted in the mind as signs, and the intensity of the emotions they contain does not change over time. Signs are attached to emotions in clinical situations. Words cannot keep up with this semiotic activity.

The referents that form the self in Umwelt, semiosphere are diverse and largely unacknowledged. Of course, the predominant referents for people are others. Through seeing oneself from the perspective of the Other, a rich Me dimension is created, and the self is formed through a dynamic process with I. The process is supported by the relationship of affecting and being affected, and the experience of this relationship is accumulated in layers. On the other hand, there can be crisis situations in the environment where the Other ceases to function as a referent. Within the semiosphere, the Other occupies a special position. However, the Other does not merely bestow a point of view but is a being with a body and with feelings and desires. They can be rejected and even violated.

How does one search for an alternative referent to the Other in the course of one's life? A typical example would be playground equipment for children. For music lovers, it may be a treasured musical instrument; for car enthusiasts, above all, a beloved car; for railway enthusiasts, an unseen vehicle; the list goes on and on.

## Cultivating the Body’s Sense of Interoception

For von Fircks’ (2023a) conclusion that mindfulness meditation itself follows a socially semiotic mediated process to hold, it must be clear which points in the sign-space are being activated. The clue in this regard is the intersection of the inner environment, i.e. the living body, in Umwelt. In this regard, it is noteworthy that Mead states that the environment also exists under the skin, i.e. in the body. Some stimuli are within the organism itself, but these stimuli are nevertheless part of the environment. Respiration is due to the presence of salts in the blood and glandular fluid, for example. The environment is not only outside the skin, but also under the skin (Mead, 1934/2015).

In respiration, oxygen enters the bloodstream = environment and enters the cells in various ways. The body is in the environment, but the environment is also in the body. In other words, the self acts on the environment through the body, and is moved and affected or sensitized. The lived body is intrinsically sensed from within and deepened as an experience. This is the very process of mindfulness meditation practice. Pay attention to the breath. Cultivating the interoceptive sense of the body is part of this practice.

Interoception is a concept that refers to the sensation of physiological states inside the body (Fukushima, 2018). It can also be generally described as 'visceral sensation'. In the theory of interoceptive sensation, interoception includes not only the visceral system, but also physiological parameters such as water content in the body, body temperature, blood sugar and oxygen saturation, and the brain's processing of all physiological states, including the autonomic nervous system, hormonal system and immune system (Craig, 2003).

In addition to the proprioceptive senses, there are also self-receptive senses (processing of information on skeletal muscle stretch) and vestibular senses (sensation of body inclination), which are bodily senses with receptors (sensors) only in the body. The proprioceptive and vestibular senses provide a perception of the positional relationship and posture of body parts within the space of the external world, which can be described as a sense of spatial positioning of the body in relation to the outside of the body. Here, the interoceptive senses include the above and monitor the situation of the body's internal world. Appropriate bodily sensations, mainly the interoceptive senses, are a major factor contributing to our physical and mental health, wellbeing.

## Tonus

The interoceptive sensed changes in the self-body are called tonus (Janet, 1909; Morioka, 2002). Janet called the changes in body sensation felt between contraction and relaxation of muscle tension, tonus (mental tension; *tonus mental*) (Janet, 1909). Tonus is the ability of a living organism to maintain its posture or stance. This postural sense is then joined to the emotional state. The term tonus can be used for mental events in parallel with neurological events, such as nervous and emotional tonus. Tonus can be seen as a continuous tension (tonic) that gives muscles a certain shape and firmness, and as a systemic function that governs various attitudes and postures. It is distinguished from the phasic contractile activity of muscles which acts externally. Tonus is a self-receptive activity that acts on the self and is closely related to changes in emotion. Sullivan (1953) also considers changes in muscle tonus to be a systemic phenomenon throughout the major muscular systems that occur independently of direct action or movement, and that changes in muscle tonus are closely related to need fulfilment and to the sensation of pleasure or displeasure. Thus, tonus is a psychosomatic correlative concept. The body as a semiosphere can be examined using tonus as a cue for sign mediated meaning making.

## Referents with the Non-human Environment

Within this semiosphere of the human Umwelt, the other is not the only opportunity to bring about a shift in perspective in self formation. In his dialogue with Rogers, Buber advocated the existence of an I-thou dimension in which one encounters the Other with one's whole being. In fact, the encounters in which Buber's I-thou relationship is founded are by no means limited to relationships with people. He shows that the relationship can also be established with the surrounding trees, creatures and animals. Take the example at the beginning of “*I and Thou*”.

A tree is represented differently according to my attitude. I count the tree as a figure, as a movement, as an incorporation into a taxonomic tribe, as an expression of certain laws, and in all these cases it is my object. It is an 'It', an object. If I have the will to do so and at the same time receive the action of Grace, it can also happen that I am brought into a relationship with that tree while observing it. At this time, the tree is no longer 'It'. I am then caught in the power of unity (Aussichließlichkeit). This is how Buber describes the encounter with an unique tree. This can be said to represent the relational dimension in which the inherent meaning-generating of the Other for the self is fully realized in the semiosphere.

On the other hand, in order to enter into this relationship, Buber states that it is not necessary to exclude or ignore knowledge about the tree taken as an object. The shapes, movements, races, specimen classifications, laws and numbers of the trees are all inseparably congruent within that tree. Everything is wrapped up within one wholeness. In other words, there is no need to get rid of the world of objects taken as 'It'. When it is in its own unity, the tree faces me as a living being. As I relate to the tree, it also relates to me.

The theme of von Fircks (2023a), that meditation, which at first glance is an internal meditation discipline and a personal activity, can also be a semiotic mediated social process, needs to be examined against this background. The reality of encountering the new I through mindfulness meditation is likely to be clarified in practice by placing the encounter with the Other in Umwelt and its semiotic activities in the background. The process of encountering the new I and regenerating as a person through mindfulness meditation emerges in its positive meaning only in the non-human environment rather than in the sphere of self and other relations. And Tao's worldview relies not only on person-to-person interactions, but also on interactions with nonhumans. von Fircks (2023b) overcomes the conventional theoretical framework approaches the way to leadership from the perspective of multicultural understanding from the Tao. von Fircks (2023a) has a different perspective and background to the Western worldview on culture and nature behind his issues. Further relativization of one's own perspective must be considered.

Viveiros de Castro (1998), a leading perspectival anthropologist, states that.

We can see that between the reflexive 'I' of culture (the generator of the concepts of soul or spirit) and the impersonal 'it' of nature (definer of the relation with somatic alterity), there is a position missing, the 'you', the second person, or the other taken as other subject, *whose point of view is the latent echo of that of the* 'I'. I believe that this concept can aid in determining the supernatural context. An abnormal context wherein a subject is captured by another cosmologically dominant point of view, wherein he is the 'you' of a non-human perspective, Supernature is the form of the Other as Subject, implying an objectification of the human I as a 'you' for this Other (Viveiros de Castro, 1998, p.483; my emphasis).

The perspective that assumes personhood through standard European language must also be put on hold for once, and a shift in mode of thought is required. Here, the position of the second person, i.e. taking the Other as the subject, and the perspective from that position as a potential echo of the 'I' perspective, is echoed in the author's mindfulness meditation practice process, where the perspective of the Other leads to the generation of a new I. And in the process, the otherness is not only the human, but also the 'you' of the non-human perspective. The supernatural in mindfulness meditation, the flow of *Ki* through the breath, comes in the form of the Other as subject. Through the perspective of this Other, a new 'I' of the Human rises up. The self-other relationship that emerges is complex but brings a rich future.

## Conclusion

This article supplements and connects different perspectives on the meaning of the self-generating process of mindfulness meditation practice to a richer discussion. By capturing Mead's theory of the self against the background of eco-environmentalism, a form of theorizing that includes the body's internal environment, it also seems to intersect with Tao's cosmology. The processual nature of the self, the characteristic of being a relational being, emerges through mindfulness meditation. As von Fircks describes in his autoethnography, the new I is actually born. Its reality is important.

Mead theorized a path of generative change of the self in relation to others and the environment, the internal environment of the body. How does mindfulness meditation work as a semiotic mediator and set in motion the process of meaning making in relation to the constitution of the new I, which until then was implicit? von Fircks' question, I feel, starts from a sense of crisis in which the other is no longer working adequately as a referential body to produce the self in the present age. Through mindfulness meditation, he focuses on the body and breath, which can be directly collated, and places the starting point for the creation of the ego there. This study seems to contain a strong message of starting from the point of directly sensing the rhythm of tension and relaxation in the body that can be felt from within.

## Data Availability

No datasets were generated or analysed during the current study.
